# Sex‐specific associations of the maturation locus *vgll3* with exploratory behavior and boldness in Atlantic salmon juveniles

**DOI:** 10.1002/ece3.11449

**Published:** 2024-06-04

**Authors:** Paul Bai Bangura, Katriina Tiira, Tutku Aykanat, Petri T. Niemelä, Jaakko Erkinaro, Petra Liljeström, Anna Toikkanen, Craig R. Primmer

**Affiliations:** ^1^ Organismal & Evolutionary Biology Research Program, Faculty of Biological and Environmental Sciences University of Helsinki Helsinki Finland; ^2^ Lammi Biological Station, Faculty of Biological and Environmental Sciences University of Helsinki Helsinki Finland; ^3^ Natural Resources Institute Finland (Luke) Oulu Finland; ^4^ Institute of Biotechnology, Helsinki Institute of Life Science (HiLIFE) University of Helsinki Helsinki Finland

**Keywords:** Atlantic salmon, behavioral syndrome, evolutionary trade‐offs, maturity age, vgll3 genotype

## Abstract

Studies linking genetics, behavior and life history in any species are rare. In Atlantic salmon (*Salmo salar*), age at maturity is a key life‐history trait and associates strongly with the *vgll3* locus, whereby the vgll3*E allele is linked with younger age at maturity, and higher body condition than the vgll3*L allele. However, the relationship between this genetic variation and behaviors like boldness and exploration which may impact growth and reproductive strategies is poorly understood. The pace‐of‐life syndrome (POLS) framework provides predictions, whereby heightened exploratory behavior and boldness are predicted in individuals with the early maturation‐associated *vgll3* genotype (*EE*). Here, we tested these predictions by investigating the relationship between *vgll3* genotypes and exploration and boldness behaviors in 129 juveniles using the novel environment and novel object trials. Our results indicated that contrary to POLS predictions, vgll3*LL fish were bolder and more explorative, suggesting a genotype‐level syndrome including several behaviors. Interestingly, clear sex differences were observed in the latency to move in a new environment, with vgll3*EE males, but not females, taking longer to move than their vgll3*LL counterparts. Our results provide further empirical support for recent calls to consider more nuanced explanations than the pace of life theory for integrating behavior into life‐history theory.

## Graphical abstract

This study investigates the relationship between the vgll3 genotype and behaviors like boldness and exploration in Atlantic salmon (*Salmo salar*). It challenges the pace‐of‐life syndrome (POLS) predictions by revealing that salmon with the late maturation genotype (vgll3*LL), contrary to expectations, displayed greater boldness and exploratory behavior. The study also highlights sex‐specific differences in these behaviors, extending the understanding of behavioral syndromes to include genetic factors linked to life‐history traits.

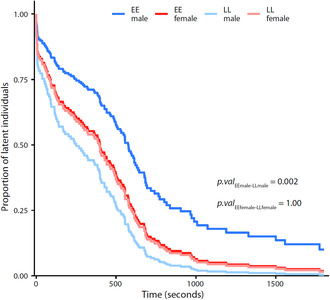

## 1 INTRODUCTION

The resources for life history traits such as age and size at maturity and number of offspring must be obtained from the energy budget of an organism, and thus are subject to trade‐offs. As such life‐history traits are generally heritable, understanding the factors contributing to the evolution of life‐history strategies is a central focus in biology (Roff, 2002; Stearns, 1992). Behavior can affect the expression of life‐history traits, as can sex‐specific differences, which add additional layers of complexity to their genetic underpinnings (Hämäläinen et al., 2018). Behavioral traits, such as boldness and exploration, can affect life history strategies by influencing an organism's interactions with its environment and thereby resource acquisition (Dingemanse et al., 2007; Réale et al., 2007). Moreover, the genetic architecture of these behavioral traits can further modulate their impact on life history variation (Laskowski et al., 2021; Sih et al., 2004a; Wolf & Weissing, 2012).

Genetic and genomic studies have revealed that 20% to 50% of the phenotypic variation in animal behavioral traits has a genetic basis (Dochtermann et al., 2015; Van Oers et al., 2008). This genetic basis could link behavioral expression with life history, as indicated in one of the key hypotheses explaining why individual variation in behaviors and physiological traits exists (Dammhahn et al., 2018; Réale et al., 2010). In this pace‐of‐life (POL) framework, one of the most studied life‐history continua is the fast‐slow continuum which can explain as much as 70% of life‐history variation among animal species (Healy et al., 2019). This framework was also expanded to explain behavioral variation within species and populations so that life‐history variation explains behavioral variation in a predictable manner (Dammhahn et al., 2018; Laskowski et al., 2021; Montiglio et al., 2018). According to this framework, termed the pace‐of‐life syndrome (POLS), early maturing individuals are predicted to be more explorative and active in maintaining their “fast” life history strategy but might be more vulnerable to predation due to their “risky” behavior, compared to late maturing individuals. However, while it has been suggested that more than 50% of variation in animal personality variation may be explained by additive genetic variation (Dochtermann et al., 2015), knowledge of the specific loci underlying the heritable genetic component of such behavioral traits is less well understood (Bubac et al., 2020).

Atlantic salmon (*Salmo salar*) is an ideal study species to increase our understanding of links between genetics, behavior, and life‐history variation. Age at maturity in Atlantic salmon affects fitness traits including survival, size at maturity, and reproductive fitness, resulting in an evolutionary trade‐off whereby larger, later‐maturing individuals have higher reproductive fitness but also have a higher risk of death prior to reproducing (reviewed by Mobley et al., 2021). Importantly, the genetic basis of age at maturity has been well characterized in Atlantic salmon, with a single nucleotide polymorphism (SNP) at the transcription co‐factor vestigial‐like family member 3 (*vgll3*) locus explaining 39% of the observed variation in sea age of maturity of males and females (Barson et al., 2015). The mechanisms by which *vgll3* genotype influences age at maturity are not fully understood, although several studies have begun to shed light on this topic. For example, juvenile Atlantic salmon harboring the allele linked with earlier maturation (vgll3*E) display higher body condition than individuals with the vgll3*L allele (later maturation allele) (Debes et al., 2021). This was hypothesized to mediate earlier maturation in vgll3*EE individuals via their having larger fat reserves available for gonad development (Debes et al., 2021). We recently tested the hypothesis that higher body condition may be mediated via vgll3*EE juveniles exhibiting more aggressive behavior than their vgll3*LL counterparts, thus giving them higher access to food (Bangura et al., 2022). Contrary to this prediction, the opposite was found: individuals with the vgll3*LL genotype were significantly more aggressive than vgll3*EE genotype individuals. Further, highly aggressive juveniles were also lighter in color and had significantly higher feeding activity, but neither of the two latter traits was significantly associated with *vgll3* genotype (Bangura et al., 2022). In a parallel study, Prokkola et al. (2022) revealed a link between *vgll3* genotype and juvenile salmon physiology, with the *vgll3* early maturation genotype being associated with a higher maximum metabolic rate and broader aerobic scope.

The abovementioned results imply that aggressive behavior may not always be beneficial in terms of growth and/or body condition, for example, in a scenario in which food cannot be monopolized, due to energy loss caused by increased movement or conflict‐related injuries cannot offset the energy gain (Ang & Manica, 2010; Bangura et al., 2022). It was therefore concluded that in the context of the experimental conditions, there was no clear evidence that the higher body condition previously linked to the vgll3*EE genotype was mediated via vgll3*EE individuals being more aggressive (Bangura et al., 2022) or having a broader aerobic scope (Prokkola et al., 2022), but rather, aggression could potentially reduce body condition in vgll3*LL individuals. In other words, it is possible that increased aggressiveness in Atlantic salmon may lead to either net energy loss or net energy gain, depending on the ecological context (Bangura et al., 2022; Jakobsson et al., 1995; Réale et al., 2010). Therefore, additional studies are required to determine if the association observed between aggressiveness and *vgll3* genotype extends to other behavioral traits.

Considering the pace‐of‐life syndrome in the context of Atlantic salmon maturation, it could be hypothesized that earlier and later maturing individuals/genotypes would constitute ‘fast’ and ‘slow’ pace of life strategies, respectively, given that earlier maturing individuals also have lower reproductive success and die earlier (see above). However, as noted above, earlier results related to aggressive behavior and physiology are not completely in line with POLS predictions, with likely later maturing vgll3*LL individuals being more aggressive (Bangura et al., 2022) but having reduced aerobic scope (Prokkola et al., 2022) compared to vgll3*EE individuals. Therefore, as noted by Laskowski et al. (2021), more research for determining the role of behavior in resource allocation and acquisition is required.

Here, we investigate behavioral traits that were previously suggested to be linked via the pace of life syndrome framework (Hall et al., 2015; Le Galliard et al., 2013) and test whether Atlantic salmon juveniles with alternative *vgll3* genotypes differ in their behavioral phenotypes in a manner consistent with our previous study (Bangura et al., 2022), or whether they vary according to POLS predictions. Fast pace life individuals may increase their fitness by being bolder and taking more risks, while slow individuals tend to avoid such risks and/or invest in different fitness components (current vs. future reproduction) (Wolf & Weissing, 2010; Wright et al., 2019). Boldness and exploratory behaviors have been suggested to provide fitness advantages in fish (Wilson & Nussey, 2010), but may be influenced by other factors such as food availability (Holley et al., 2014) and temperature (Angiulli et al., 2020). Individuals exhibiting explorative behaviors may be rewarded by increased offspring production but may be also more prone to predation. On the other hand, shyer individuals may live longer and have higher lifetime reproductive success than more aggressive conspecifics (reviewed by Laskowski et al., 2021).

Studying whether behavior traits that reflect boldness and exploration are associated with the *vgll3* genotype allowed us to make two alternative predictions: according to the POLS, faster and higher exploration was predicted in vgll3*EE individuals (the earlier maturing, “fast” phenotype) than later maturing vgll3*LL individuals (the “slow” phenotype). However, our earlier results related to aggressive behavior were in the opposite direction to POLS predictions, with slow phenotype vgll3*LL individuals being more aggressive (Bangura et al., 2022), this leads to the alternative prediction that a genotype‐driven behavioral syndrome (albeit not in line with the POLS specifically) is maintained, whereby vgll3*LL individuals would also be bolder and more exploratory. Here, we investigate the association between exploratory behaviors and *vgll3* genotype to test these predictions in juvenile Atlantic salmon.

## 2 METHODS

### 2.1 Experimental animals

The Atlantic salmon juveniles used in this study were full siblings of those used in Bangura et al. (2022). They were derived from a first‐generation hatchery broodstock of salmon managed by the Natural Resources Institute Finland (LUKE). The parents were crossed in October 2019 to create 14 *vgll3* homozygote full‐sib families (seven vgll3*EE families and seven vgll3*LL families). We used homozygous families to maximize sample sizes of individuals with genotypes expected to exhibit the largest phenotypic differences associated with *vgll3*. Fertilized eggs were incubated in replicated, family‐specific, compartments at a stable water temperature (average of 7.2°C) until March 2020.

After hatching, alevins were transferred to the experimental facilities at Lammi Biological Station (61°04′45″ N, 025°00′40″ E, Lammi, Finland), several weeks before they commenced independent feeding. Each family (60–200 individuals per family) was reared in a randomly selected separate tank (90 cm diameter) as outlined in Bangura et al. (2022). Individuals were exposed to ambient lighting conditions, with fluorescent lights evenly distributed above the tanks at a height of 50 cm, reflecting the local photoperiod that transitioned from 10 hours of light and 14 h of darkness at the start to 18 h of light and 6 h of darkness by the experiment's end. The water temperature was 4.5°C when the transfer to Lammi Biological Station was made and rose gradually from an average of 4.7°C in March to 5.7°C in May when the behavioral experiments were conducted (see Åsheim et al., 2022 for temperature curve). The average mass of individuals used in trials was 0.31 g (measured using the Ohaus Scout Pro scale with a precision level of 0.01 g) and the average length was 3.3 cm. The *vgll3* genotypes of families and individuals were unknown to people participating in fish farming and to those conducting the behavioral experiments and were revealed after the behavioral data had been finalized.

### 2.2 Behavioral trial setup

Behavioral trials were conducted in twenty‐six identical aquaria each measuring 30 cm in width, 25 cm in depth, and 40 cm in height. Each aquarium was filled to a depth of 30 cm and had a flow rate of approximately 3 L/min, resulting in standardized environmental conditions across all experimental units. A floating feed ring (5 cm) was placed on the water surface in the middle section of each aquarium so that food pellets would drift along with the water current, thereby further enforcing a profitable territory location. Environmental enrichments were not provided in the experimental aquaria to allow better observation of fish during the trial. The aquaria were covered on three sides to minimize disturbance, and the top was covered with polystyrene to prevent the fish from jumping from the aquaria. The photoperiod and water source were the same as those of the holding tanks described above. All 26 aquaria were used concurrently in a trial round (resulting in 1–2 individuals from each of the 14 families being included in each trial round), with the experiment including a total of five trial rounds including 26 individuals, and thus 130 individuals in total. The individuals had no prior exposure to their respective aquaria before the experiment, and the experimenters were unaware of the *vgll3* genotypes of these individuals. The initial water temperature was documented at the onset of each trial. No deaths or apparent harm were recorded among the fish used in the trials throughout the experiments. After completing each trial round, individuals in each of the 26 aquaria were removed and humanely euthanized using an overdose of sodium bicarbonate‐buffered methanesulfonate (250 mg/L). A fin sample was collected and preserved in 95% ethanol for the purpose of genetically confirming the *vgll3* genotype and sex, which were done using Kompetitive allele‐specific polymerase chain reaction (KASP) assays (He et al., 2014) for the *vgll3*_TOP_ SNP and the sex‐specific SDY locus. The reaction mix for each reaction consisted of 2.5 μL of sample DNA, 2.5 μL KASP 2× Master mix, and 0.07 μL KASP Assay mix which contains the locus‐specific primers. The reactions were performed with quantitative PCR (qPCR) machines (C1000 Thermal cycler with CFX384 Real‐Time System; Bio‐Rad). Genotypes of the *vgll3* SNP were called using allelic discrimination implemented in the CFX Maestro software (Bio‐Rad). Genotypic sex was determined by analyzing the per‐individual difference between ROX‐standardized FAM and HEX fluorescence values. See Sinclair‐Waters et al. (2022) for more details and primer sequences.

### 2.3 Behavioral trials

**FIGURE 1:**
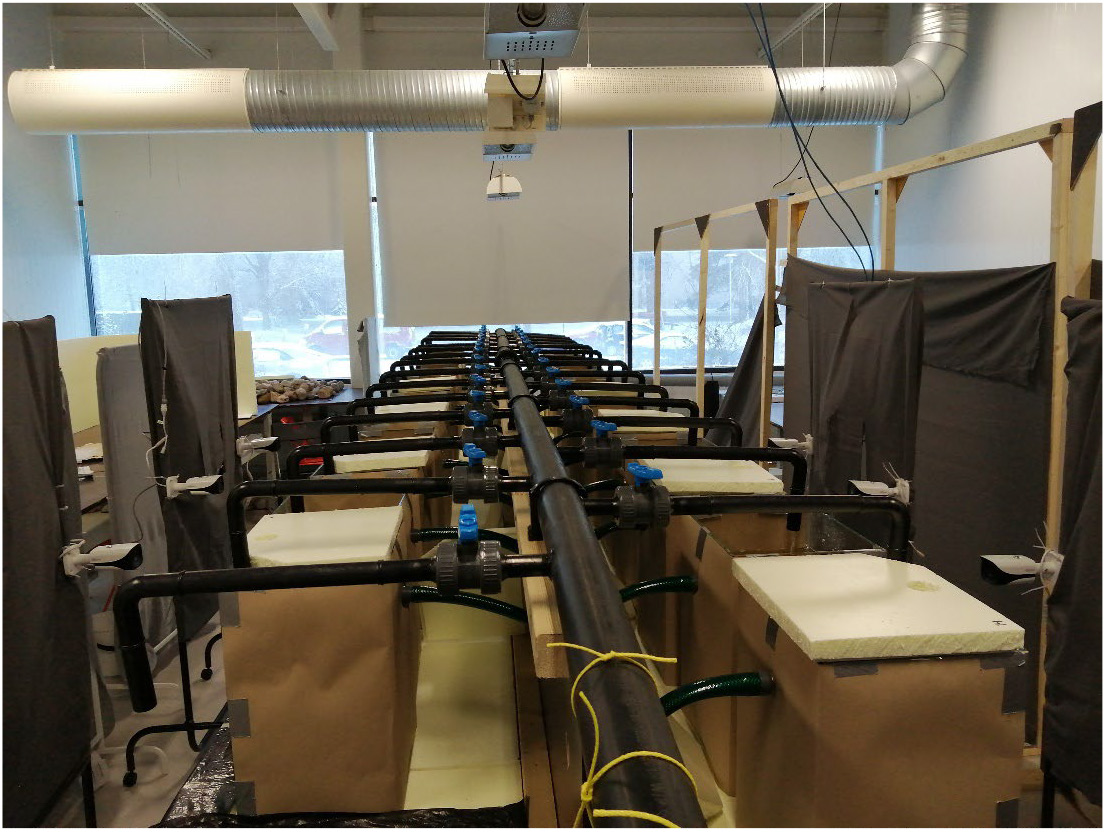
Image of the experimental setup with cameras positioned in front of each aquarium.

We quantified exploration and boldness behaviors separately, using the novel environment and novel object tests, respectively. Novel environment tests were conducted 24 h after feeding was stopped and the focal fish were isolated from their siblings. Single fish were randomly netted out of their rearing tank and placed into each of the 26 aquaria and their behavior exploring the new environment was recorded for 30 min using Dahua video surveillance cameras (Figure 1). At the end of the recording period, each individual remained alone in its aquarium without a feed until the novel object test was conducted the following day (see below).

Novel object tests, aiming to assess individual boldness, were conducted on the same individuals the following day. At the start of the (video recorded) observation period of 15 min, fish were fed 0.5 g of pellet food (0.5 mm) and simultaneously presented with a novel object in the form of an orange 7 cm fishing jig, a type of fishing lure resembling a larva/worm made from rubber that was gently lowered to the bottom of the aquarium in the same region of the aquarium where the food was released. The activity of each fish was then video recorded (see above) for 15 min for later analysis.

During the trials, juvenile salmon mostly assumed positions near the aquarium's center, orienting themselves against the flow of the current. Despite the salmon exhibiting movement throughout, their movements were mainly motivated by the pursuit of drifting pellet food. Upon successful feeding, the individuals typically reverted to their initial positions in the central area, demonstrating a pattern of consistent foraging behavior (e.g.Wankowski & Thorpe, 1979).

### 2.4 Behavioral measurements and data collection

The video recordings for the novel environment test were manually analyzed by an observer using a stopwatch, while those of the novel object test were analyzed using Ethovision XT15 software (Noldus IT, Wageningen, Netherlands). In the latter case, event rules were configured to accurately and consistently measure different behaviors within the testing arena. Overall, 130 fish (five trial rounds, each including 26 fish) were observed. However, the accidental loss of one video file left 129 fish including 62 males and 67 females (vgll3*EE = 67 (Female: 37, Male: 30) and vgll3*LL = 62 (Female: 30, Male: 32**)** individuals) for further analysis.

In the novel environment test, two variables were derived from the videos to measure exploration activity. *Latency to the first movement*, where shorter latency implies higher boldness, was measured as the time taken (in s) for a fish to move for the first time after it was introduced in the aquarium, and *time spent moving* was calculated as the total duration (in s) of swimming activity during the 30 min trial period (Kotrschal et al., 2014, Lucon‐Xiccato & Bisazza, 2017) with one fish body length/second used as a threshold for movement, allowing us to control for individual length size. The response variable “percent moving time” in the exploration novel object trials were used to allow comparison of the novel environment and novel object trials, which were different lengths, and were calculated by dividing the moving time by the total trial time for each fish. In the small number of cases where a fish did not move during the trial, this was recorded as zero percent. These same two variables were also derived for the novel object trials, as well as two additional variables: “*Entered the jig zone*” which was recorded as “yes”if an individual entered the jig zone (defined as a 12 cm radius around the jig) at any time during the trial or “no” if it did not enter the jig zone at any time during the 15 min strial period. “*Feeding activity*” was the number of food items eaten by a fish during the 15 min trial period.

### 2.5 Statistical analysis

All analyses were performed in R v. 4.2.3 (R Core Team, 2018) using the RStudio (RStudio Team, 2015) environment. The following packages were used: *dplyr* 1.1.0 (Wickham et al., 2019), *glmmTMB 1.1.5* (Brooks et al., 2017), *effects 4.2.2* (Fox et al., 2016), *emmeans* 1.8.5 (Lenth & Lenth, 2018), *coxme 2.2.18.1* (Therneau, 2022), *DHARMa 0.4.6* (Hartig & Hartig, 2017), *ggplot2 3.4.3* (Wickham et al., 2016), survival 3.5–5 (Therneau & Therneau, 2015).

We employed a generalized linear mixed effect model for each of the response variables, with differing residual error distribution depending on the type of the response variables (Table 1). In all models, the factor structure was as follows:

$$Y=\mu +\text{vgll3}+\mathrm{sex}+\text{vgll3}:\mathrm{sex}+L+\mathrm{CF}+\varepsilon_{\text{trial}}+\varepsilon_{\text{family}}+\varepsilon_{\text{aquarium}}+\varepsilon_{\text{error}}$$

whereby, *Y* is the response variable (Table 1), *vgll3* is the individual's genotype at the *vgll3* locus (EE (linked with early maturation age) or LL (linked with late maturation age)), sex is the genetically determined sex of the individuals, and L and CF are Length and Fulton's condition factor (Nash et al., 2006) measured after the trial period. ε_trial_, ε_family_, ε_aquarium_ are variances associated with random effects of trial round, family origin of the individual, and the aquarium ID that an individual was placed in, respectively. The random effects were balanced and uncorrelated and we employed an unstructured variance‐covariance structure in the model. For time‐to‐event variables (*Latency to the first movement*), we employed a Cox regression (survival model) using the *coxme* function in the *coxme* package (Therneau, 2012), whereby the time‐to‐event is modeled with respect to the total time of the experiment. We employed beta‐regression in the glmmTMB package, for the *Time spent moving* variable. We employed a negative binomial model in the glmmTMB package for the Boolean *Entering the jig zone* and *feeding activity* variables.

**TABLE 1:** Behavioral variables were measured for both exploration and novel object test and their models.

Test variable	Definition	Behavioral test	Model
Latency to the first movement ‐ Novel Environment	The time is taken for a fish to move for the first time after being introduced to the aquarium (in s)	Novel environment	Cox model (survival analysis) using coxme function
Latency to the first movement ‐ Novel Object	The time taken for a fish to move from its initial position after the jig was introduced (in s)	Novel object	Cox model (survival analysis) using coxme function
Time spent moving‐novel environment	The percent of the total trial time (30 min) spent moving	Novel environment	Beta regression model using beta family with log link in glmmTMB
Time spent moving‐ novel object	The percent of the total trial time (15 min) spent moving	Novel object	Beta regression model using beta family with log link in glmmTMB
Entered the jig zone	Whether or not an individual entered the jig zone during the 15 min trial (yes/no)	Novel object	Binomial model with log link in glmmTMB
Feeding activity	The number of food pellets eaten by a fish during the 15 min trial	Novel object	Negative binomial model in glmmTMB

The model described above modeled the sex and *vgll3* genotype interaction (i.e. interaction model). We also tested an additive model that does not contain the interaction term between the two and a null model without the *vgll3* effect. We compared the fit of these alternative models using the Akaike Information Criterion (AIC). Diagnostics for the models were inspected by comparing simulated and observed residuals using the *R* package *DHARMa* for models that were fit using the *glmmTMB* package. Violations for the proportional hazards assumption of cox regressions were tested using the *cox.zph* function in the survival package in R. Marginal means, and *p*‐values were obtained using the *emmeans* package.

## 3 RESULTS

During the novel environment tests, the average latency to the first movement of an individual was 447.16 s (range 0–1800) and individuals moved for an average of 229.74 s (12.8%) of the 30‐min (1800 s) trial period (range 0–1372). In the novel object tests, the average latency of an individual to move following the introduction of the novel object (fishing jig) was 218.9 s. (range 0.72–880.5) and individuals moved for an average of 221.4 s (24.6%) of the 15 min (900 s) trial period (range 3.8–894.4). The distributions of the behavioral traits used as response variables are shown in Appendix 1.

**FIGURE 2:**
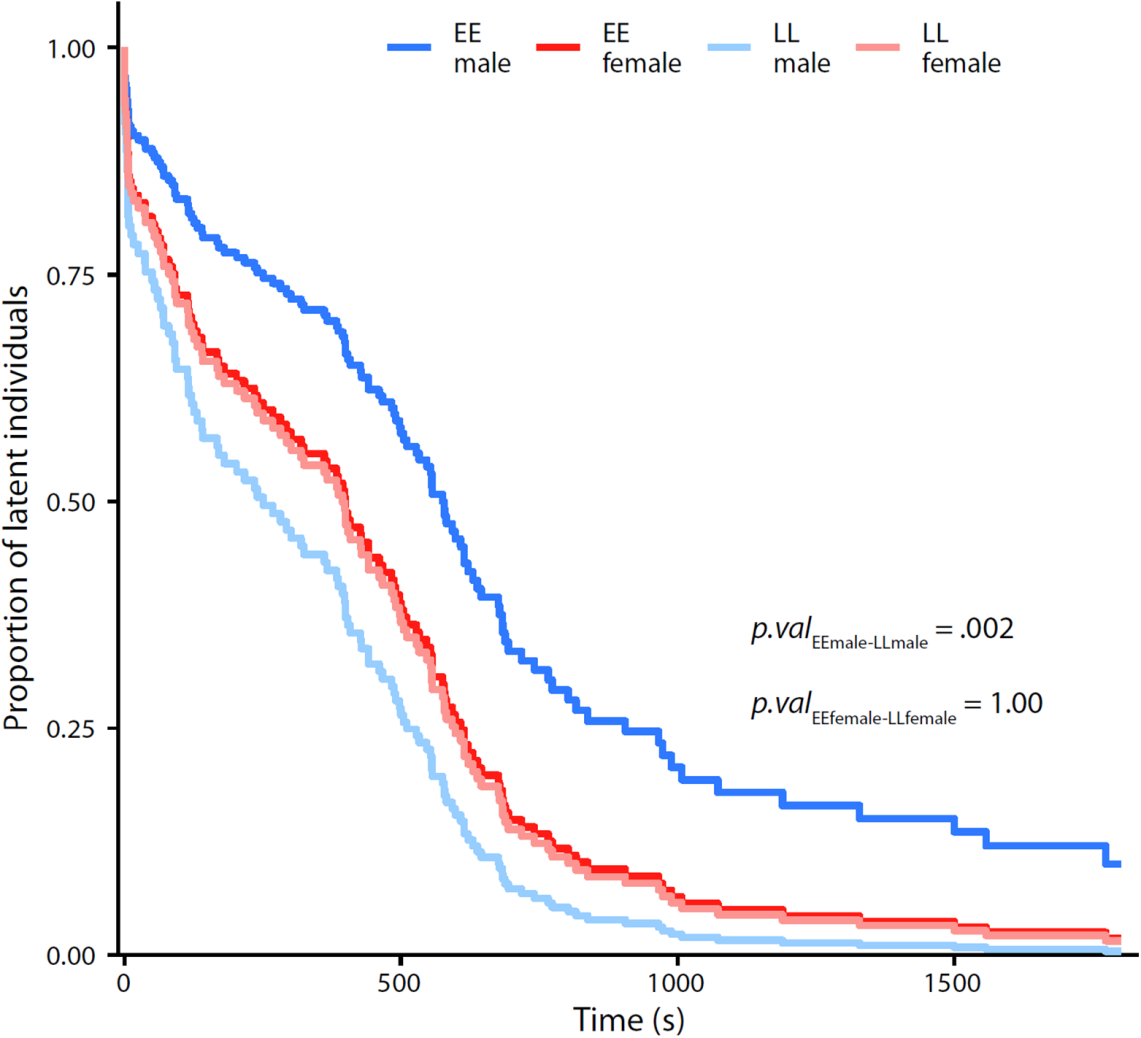
Interaction model of the cumulative proportion of latent males (blue)and females (red) with alternative vgll3 genotypes during the 30 min ‘Novel environment’ trial.

Latency to first move in the novel environment tests revealed a significant *vgll3* genotype by sex interaction (Table 2, Figure 2). More specifically, there was a significantly (*p* = .002) higher proportion of vgll3*EE males that remained latent at any given time in the trial than there were vgll3*LL males, while the proportion of latent female individuals was consistently intermediate (higher than vgll3*LL males, but lower than vgll3*EE males), with no significant difference between *vgll3* genotypes in females (*p* = 1, Figure 2). Both the genotype and sex effect were diminished for the latency to first move in the novel object trial, whereby the null model was the most parsimonious one.

**TABLE 2:** Coefficient estimates (E, plus Standard Error – SE) from linear mixed‐effects and Cox regression models testing the effects of vgll3 genotype and sex, and their interaction on six behavioral variables.

	Additive model				Interaction model				Null model			
	Est.	SE	*z*	*p*	Est.	SE	*z*	*p*	Est.	SE	*z*	*p*
Latency first move‐exploration test												
Genotype (LL)	0.492	0.211	2.337	.334	−0.0009	0.287	−0.003	.997				
Sex (Male)	−0.193	0.200	−0.966	.302	−0.791	0.301	−2.621	.009	−0.251	0.197	−1.272	.203
Length	0.386	0.374	1.032	.538	0.457	0.378	1.208	.227	0.652	0.350	1.865	.062
Condition factor	−0.902	1.466	−0.616	.019	−1.397	1.516	−0.921	.357	−0.845	1.449	−0.578	.561
Genotype(LL):sex (Male)	–	–	–	–	1.092	0.411	2.658	.008	–	–	–	–
AIC	978.8				971.7				983.5			
Time spent moving‐Exploration test												
Genotype (LL)	0.538	0.176	3.055	.002	0.406	0.235	1.723	.085	–	–	–	–
Sex (Male)	−0.357	0.168	−2.122	.034	−0.510	0.247	−2.063	.039	−0.382	0.175	−2.186	.029
Length	0.802	0.336	2.386	.017	0.827	0.339	2.438	.015	1.073	0.340	3.160	.002
Condition factor	−1.960	1.287	−1.523	.128	−2.017	1.277	−1.579	.114	−1.734	1.304	−1.330	.184
Genotype(LL):sex (Male)	–	–	–	–	0.275	0.327	0.842	.400	–	–	–	–
AIC	−287.76				−286.46				−280.65			
Latency first move‐Novel object test												
Genotype (LL)	0.129	0.237	0.546	.584	0.431	0.309	1.397	.162	0.51	0.203	2.507	.012
Sex (Male)	0.498	0.204	2.445	.014	0.831	0.293	2.841	.004	−0.374	0.376	−0.995	.319
Length	−0.438	0.393	−1.115	.264	−0.437	0.340	−1.093	.274	3.211	1.583	2.029	.042
Condition factor	3.200	1.580	2.025	.042	3.675	1.602	2.293	.022	–	–	–	–
Genotype(LL):sex (Male)	–	–	–	–	−0.626	0.401	−1.562	.118	–	–	–	–
AIC	957.33				956.22				956.09			
Time spent moving‐Novel object test												
Intercept	−2.810	1.699	−1.654	.098	−2.797	1.699	−1.646	.100	−3.284	1.671	−1.965	.049
Genotype (LL)	0.299	0.200	1.495	.135	0.261	0.278	0.942	.346	–	–	–	–
Sex (Male)	0.300	0.193	1.559	.119	0.263	0.273	0.964	.335	0.313	0.193	1.617	.106
Length	0.054	0.366	0.149	.882	0.056	0.366	0.153	.879	0.214	0.352	0.608	.543
Condition factor	1.807	1.472	1.228	.220	1.806	1.472	1.227	.220	1.915	1.468	1.305	.192
Genotype(LL):sex (Male)	–	–	–	–	0.074	0.381	0.194	.846	–	–	–	–
AIC	−73.89				−71.92				−73.67			
Feeding activities‐Novel object test												
Intercept	−1.321	3.528	−0.374	.708	−1.415	3.644	−0.388	.697	1.650	3.417	−0.483	.629
Genotype (LL)	0.176	0.443	0.398	.690	0.788	0.6537	1.205	.222	–	–	–	–
Sex (Male)	0.287	0.414	0.634	.487	0.878	0.629	1.394	.163	0.288	0.412	0.699	.484
Length	−0.212	0.783	−0.271	.786	−0.304	0.804	−0.378	.704	0.102	0.730	−0.140	.888
Condition factor	1.296	3.003	0.431	.666	1.400	3.122	0.449	.653	1.362	2.987	0.456	.648
Genotype(LL):sex (Male)	–	–	–	–	−1.163	0.894	−1.301	.193	–	–	–	–
AIC	704.613				704.346				702.694			
Frequency entering Jig zone‐Novel object test												
Intercept	−1.321	3.528	−0.374	.708	−1.416	3.645	−0.388	.698	−1.651	3.417	−0.483	.629
Genotype (LL)	0.176	0.443	0.398	.691	0.788	0.654	1.206	.228	–	–	–	–
Sex (Male)	0.287	0.414	0.694	.488	0.878	0.630	1.395	.163	0.288	0.412	0.699	.484
Length	−0.212	0.783	−0.271	.786	−0.305	0.804	−0.379	.705	−0.103	0.731	−0.141	.888
Condition factor	1.296	3.003	0.431	.666	1.401	3.120	0.449	.653	1.362	2.987	0.456	.648
Genotype(LL):sex (Male)	–	–	–	–	−1.164	0.894	−1.301	.193	–	–	–	–
AIC	163.83				164.00				161.99			

*Note*: The parsimonious model with the lowest AIC is presented in this table.

Abbreviations: AIC, Akaike Information Criterion; E, estimates; *p*, probability as *Pr*(>|*z*|); SE, Standard Error; *z*, test statistics.

**FIGURE 3:**
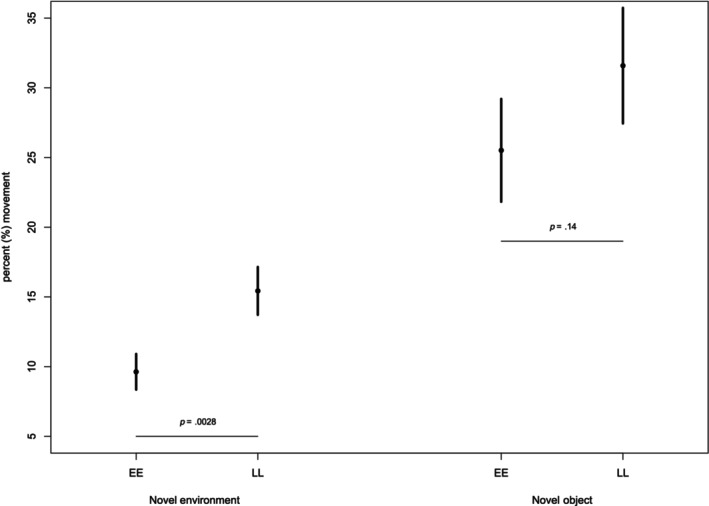
Percent of time spent moving in the novel environment (30‐min trial time) and novel object (15‐min trial time) trials for vgll3*EE and vgll3*LL genotype individuals based on the “genotype” model.

Considering the time spent moving, the additive model was the most parsimonious in both the novel environment and Novel object trials (Table 2, and Figure 3). Both the *vgll3* genotype and the sex effect were significant in the novel environment trial (*p* values .002, .034, respectively), whereby vgll3*LL individuals were significantly more active in the 30‐min period following their release to the novel environment (15.4% of time spent moving vs. 9.6% for vgll3*EE individuals; *p* = .003, Table 2, Figure 3), and females were more active than males (females were moving 14.3% of the time (95% CI = 11.3–17.8) compared to males who moved 10.4% (95% CI = 8.81–13.3) of the time (*p* = .034)).

In the novel object test, while the additive model was the best model, the null model was equally parsimonious (i.e. within AIC = 2 to the best model), and neither *vgll3* genotype nor sex effects were significant (*p* = .135 and *p* = .119, Figure 3). The level of activity was generally higher in the novel object than in the novel environment trial and the trend in the difference of time spent moving between *vgll3* genotypes was similar to that observed in the novel environment trial, but no longer statistically significant (vgll3*LL 31.5% and vgll3*EE 25.5%, *p* = .14, Table 2, Figure 3). For the feeding activity and the frequency entering the Jig zone, we did not find any support for an effect of sex or genotype, nor their interaction (Table 2).

## 4 DISCUSSION

We investigated whether the *vgll3* genotype is associated with boldness and exploration in juvenile Atlantic salmon. We aimed to distinguish between two alternative predictions: according to the POLS, faster and higher exploration was predicted in vgll3*EE individuals, while our earlier results related to aggressive behavior provided an alternative prediction whereby if a syndrome is maintained at the genotype level, then vgll3*LL individuals were predicted to be bolder and more exploratory. The results of this study are consistent with the latter prediction. More specifically, in addition to being more aggressive (Bangura et al., 2022), vgll3*LL individuals displayed higher levels of boldness and exploratory behavior both in terms of reduced latency time and of a higher percentage of time spent moving in a novel environment.

These results imply that the behavior of vgll3*LL individuals follows a genotype‐driven syndrome involving several behaviors, whereby vgll3*LL individuals potentially allocate a higher amount of energy than vgll3*EE individuals to a range of proactive behaviors including aggression (Bangura et al., 2022) boldness and exploration (this study). Future studies adding a physiological dimension to this research would be beneficial, although previous physiological research has indeed shown that vgll3*EE individuals have a broader aerobic scope (Prokkola et al., 2022). Salmon individuals having late maturing genotypes have been shown in other studies to have lower body conditions (Debes et al., 2021). Thus, it can be speculated that late‐maturing individuals may have higher maintenance costs or less efficient feeding behavior, as has been demonstrated to explain differences in body condition in other studies (Orlov et al., 2006). A possible explanation is that they fail to benefit from this behavior in terms of gaining additional energy compared to vgll3*EE individuals who have higher body conditions in some contexts despite being less aggressive. This could result in vgll3*LL individuals not having enough energy for allocation into reproduction at an early age. Further, the more explorative behavior displayed by juvenile vgll3*LL individuals could potentially be costly since feeding and hiding behind rocks is an important behavior at this life‐history stage (Metcalfe et al., 1987). Future behavioral studies including *vgll3* genotype information in more realistic ecological contexts would be beneficial in this regard. For example, size of the experimental aquaria used here may have constrained the range of exploratory and boldness behavior, as the feeding territories of similar‐sized juvenile salmon in more natural conditions are typically larger (e.g. Dill et al., 1981; McNicol et al., 1985).

From an evolutionary point of view, in late‐maturing animals, proactive behavior such as exploration could conceivably be advantageous in later life stages (Langenhof & Komdeur, 2018), whereby adult vgll3*LL individuals may be better predators and/or able to better escape predation themselves. In addition, a study on foraging behavior in fish with different developmental strategies suggests that faster‐growing individuals (analogous to 1‐year‐old smolts) are more risk‐prone and bold, aligning with strategies that prioritize rapid growth and early maturation (Metcalfe et al., 1989). This contrasts with slower growers (like 2‐year‐old smolts), who exhibit more cautious behavior, likely due to strategies balancing growth with survival. Although speculative, one can draw parallels between these findings and those of this study, where bolder and more exploratory behaviors in vgll3*LL individuals might reflect a life‐history strategy focused on rapid development and early smoltification, despite potential risks (Bangura et al., 2022). In addition to physiological experiments, also further studies on individuals at different life‐history stages are required to test the above speculations. Moreover, the inefficient feeding behavior of the vgll3*LL juveniles should be tested in different contexts, ideally in more natural conditions, as well as the future advantages that vgll3*LL adults may have in feeding and escaping from predators. It is interesting to note that two recent studies did not find any significant difference in the standard metabolic rate between salmons with different *vgll3* genotypes (Åsheim et al., 2022; Prokkola et al., 2022). Indeed, a new suggested framework for the “fast‐slow” continuum emphasized the lack of a consistent association between metabolic rate and behavior and raised the possibility that behavior may play only a minor role in driving resource allocation (Laskowski et al., 2021).

The strong sex‐specific effect of the *vgll3* genotype on latency to move adds dimension compared to our previous study of aggressive behavior where no sex‐specific associations were observed (Bangura et al., 2022). More specifically, *vgll3* genotype differences in latency time were driven almost entirely by males, with vgll3*LL males being significantly faster to initiate movement than vgll3*EE males while the female proportion of latent individuals was consistently intermediate, with no significant difference between *vgll3* genotypes. Several other studies have reported sex‐specific differences related to *vgll3* genotypes. In wild salmon populations, sex‐dependent dominance in *vgll3*‐related maturation patterns has been reported in several studies, whereby the vgll3*EE allele is dominant in males, but the vgll3*LL allele is partially dominant in females (Barson et al., 2015; Czorlich et al., 2018). In more controlled, aquaculture, conditions, the influence of the *vgll3* genotype on maturation age was observed in males, but not females (Ayllon et al., 2019). Furthermore, a study of movement activity in a semi‐natural environment observed increased movement activity in migrating females with the vgll3*EE allele, whereas the vgll3*LL allele was related to increased activity in males but no association with *vgll3* genotype was observed for activity level in nonmigrating individuals (Niemelä et al., 2022). The apparent stronger influence of the *vgll3* genotype in males in our study could potentially be explained by the fact that males often mature at a younger age than females (Barson et al., 2015), and males can mature in captivity already at 1 year of age (e.g. Debes et al., 2020). Therefore, the 0.5‐year‐old individuals used in this study may be more likely to already be affected by maturation‐related *vgll3*‐influenced processes if hormonal cascades related to the maturation process had commenced in males, but not females. The higher activity of vgll3*LL migrating males (Niemelä et al., 2022) is also consistent with the shorter time to initiate movement observed in this study, despite the differing juvenile ages and experimental set‐ups used in the two studies. Future studies on different life history stages will be beneficial to clarify this aspect.

The classical definition of a “behavioral syndrome” has considered correlated behaviors in the context of an individual or species (Sih et al., 2004b). We here extend this to include also genotypes of a life‐history linked large‐effect locus. While the correlation of risk‐taking related behaviors in vgll3*LL individuals conforms to this framework, it does not comply with the classical fast‐slow life‐history continuum, or POLS whereby “fast” phenotypes, i.e., strategies in which maturation and mortality occur earlier, are predicted to be associated with riskier behaviors (Laskowski et al., 2021). POLS predicts the trade‐off between allocating resources towards current reproduction at the expense of future survival, with “fast” phenotypes acquiring more resources and immediately allocating them to current reproduction, while “slow” phenotypes are predicted to allocate more into future reproduction (Laskowski et al., 2021). However, this simplistic association of behavioral traits associated with fast versus slow life histories has been recently challenged and it has been emphasized that the relative balance between resource acquisition and allocation may be more important than resource acquisition alone (Laskowski et al., 2021). Our results provide empirical support for the need to consider more nuanced explanations for integrating behavior into life‐history theory, in particular, the resource acquisition balance versus allocation at different life‐history stages and in different ecological contexts (Bangura et al., 2022; Geiler‐Samerotte et al., 2020; Pavličev & Cheverud, 2015).

## AUTHOR CONTRIBUTIONS

**Paul Bai Bangura:** Conceptualization (equal); data curation (lead); formal analysis (lead); funding acquisition (supporting); investigation (lead); methodology (lead); project administration (equal); software (lead); visualization (lead); writing – original draft (lead); writing – review and editing (equal). **Katriina Tiira:** Conceptualization (supporting); investigation (equal); methodology (equal); supervision (supporting); writing – review and editing (supporting). **Tutku Aykanat:** Data curation (supporting); formal analysis (equal); supervision (supporting); writing – review and editing (supporting). **Petri T. Niemelä:** Formal analysis (supporting); writing – review and editing (supporting). **Jaakko Erkinaro:** Resources (supporting); writing – review and editing (supporting). **Petra Liljeström:** Investigation (equal). **Anna Toikkanen:** Investigation (equal). **Craig R. Primmer:** Conceptualization (lead); data curation (supporting); funding acquisition (lead); methodology (supporting); project administration (lead); resources (lead); supervision (lead); writing – original draft (supporting); writing – review and editing (supporting).

## CONFLICT OF INTEREST STATEMENT

The authors have no conflict of interest.

## Data Availability

Data used for this analysis and the R code of all analyses are available from Zenodo Digital Repository: https://doi.org/10.5281/zenodo.10371637.
